# Limiting adverse birth outcomes in resource-limited settings (LABOR): protocol of a prospective intrapartum cohort study

**DOI:** 10.12688/gatesopenres.13716.2

**Published:** 2022-12-19

**Authors:** Amanda Adu-Amankwah, Mrutunjaya B. Bellad, Aimee M. Benson, Titus K. Beyuo, Manisha Bhandankar, Umesh Charanthimath, Maureen Chisembele, Stephen R. Cole, Sangappa M. Dhaded, Christabel Enweronu-Laryea, Bethany L. Freeman, Nikki L. B. Freeman, Shivaprasad S. Goudar, Xiaotong Jiang, Margaret P. Kasaro, Michael R. Kosorok, Daniel Luckett, Felistas M. Mbewe, Sujata Misra, Kunda Mutesu, Mercy A. Nuamah, Samuel A. Oppong, Jackie K. Patterson, Marc Peterson, Teeranan Pokaprakarn, Joan T. Price, Yeshita V. Pujar, Dwight J. Rouse, Yuri V. Sebastião, M. Bridget Spelke, John Sperger, Jeffrey S. A. Stringer, Methodius G. Tuuli, Michael Valancius, Bellington Vwalika, LABOR Study Group

**Affiliations:** 1 Department of Obstetrics and Gynaecology, University of Ghana Medical School, Korle-Bu Teaching Hospital, Accra, Ghana; 2 Women’s and Children’s Health Research Unit, KLE Academy of Higher Education and Research, Jawaharlal Nehru Medical College, Belgaum, India; 3 Department of Obstetrics and Gynecology, University of North Carolina School of Medicine, Chapel Hill, North Carolina, 27599, USA; 4 Women and Newborn Hospital, University Teaching Hospital of Lusaka, Lusaka, Zambia; 5 Department of Epidemiology, University of North Carolina School of Medicine, Chapel Hill, North Carolina, 27599, USA; 6 Department of Child Health, University of Ghana Medical School, Korle-Bu Teaching Hospital, Accra, Ghana; 7 Department of Biostatistics, University of North Carolina, Chapel Hill, North Carolina, 27599, USA; 8 UNC Global Projects Zambia, LLC, Lusaka, Zambia; 9 Fakir Mohan Medical College and Hospital, Balasore, India; 10 Department of Pediatrics, University of North Carolina School of Medicine, Chapel Hill, North Carolina, 27599, USA; 11 Department of Obstetrics and Gynecology, Warren Alpert Medical School of Brown University, Providence, Rhode Island, 02903, USA; 12 Department of Obstetrics and Gynaecology, University of Zambia School of Medicine, Lusaka, Zambia

**Keywords:** Labor, delivery, intrapartum, postpartum, adverse birth outcomes, machine learning

## Abstract

**Background:** Each year, nearly 300,000 women and 5 million fetuses or neonates die during childbirth or shortly thereafter, a burden concentrated disproportionately in low- and middle-income countries. Identifying women and their fetuses at risk for intrapartum-related morbidity and death could facilitate early intervention.

**Methods:** The Limiting Adverse Birth Outcomes in Resource-Limited Settings (LABOR) Study is a multi-country, prospective, observational cohort designed to exhaustively document the course and outcomes of labor, delivery, and the immediate postpartum period in settings where adverse outcomes are frequent. The study is conducted at four hospitals across three countries in Ghana, India, and Zambia. We will enroll approximately 12,000 women at presentation to the hospital for delivery and follow them and their fetuses/newborns throughout their labor and delivery course, postpartum hospitalization, and up to 42 days thereafter. The co-primary outcomes are composites of maternal (death, hemorrhage, hypertensive disorders, infection) and fetal/neonatal adverse events (death, encephalopathy, sepsis) that may be attributed to the intrapartum period. The study collects extensive physiologic data through the use of physiologic sensors and employs medical scribes to document examination findings, diagnoses, medications, and other interventions in real time.

**Discussion:** The goal of this research is to produce a large, sharable dataset that can be used to build statistical algorithms to prospectively stratify parturients according to their risk of adverse outcomes. We anticipate this research will inform the development of new tools to reduce peripartum morbidity and mortality in low-resource settings.

## Introduction

Each year worldwide, nearly 300,000 women and over 5 million fetuses or neonates die during childbirth or shortly thereafter ^1– 4^. The vast majority of this burden is borne by the world’s poor; only a tiny fraction of these peripartum deaths occur outside low- and middle-income countries (LMICs) ^5^. Estimates of the worldwide burden of perinatal death demonstrate that 22% of maternal deaths and 50% of stillbirths occur in the intrapartum period and that 26% of neonatal deaths can be attributed to intrapartum events ^4, 6^. Better identification of women and fetuses at risk for intrapartum complications could lead to improved outcomes through early intervention.

The Limiting Adverse Birth Outcomes in Resource-Limited Settings (LABOR) Study is designed to exhaustively document the course and outcomes of hospitalization, labor, delivery, and the immediate postpartum period in settings where the occurrence of adverse outcomes is high. Using information obtained from this study, we seek to develop machine learning, deterministic, and statistical algorithms that prospectively stratify women and fetuses by their risk of adverse outcomes and that signal actionable intrapartum diagnoses. These novel algorithms will lay the groundwork for the development of a robust suite of tools to reduce intrapartum morbidity and mortality in low-resource settings.

## Methods

### Study design

The LABOR Study is a multi-country, prospective, observational cohort study. It was initially designed to enroll a maximum of 15,000 mother-infant pairs but this figure was modified to 12,000 after recruitment was slowed by the SARS-CoV-2 pandemic at all sites. The study has been registered prospectively with clinicaltrials.gov under identifier NCT04102644 and with the Clinical Trial Registry of India under CTRI/2020/01/022589.

**Figure 1.**
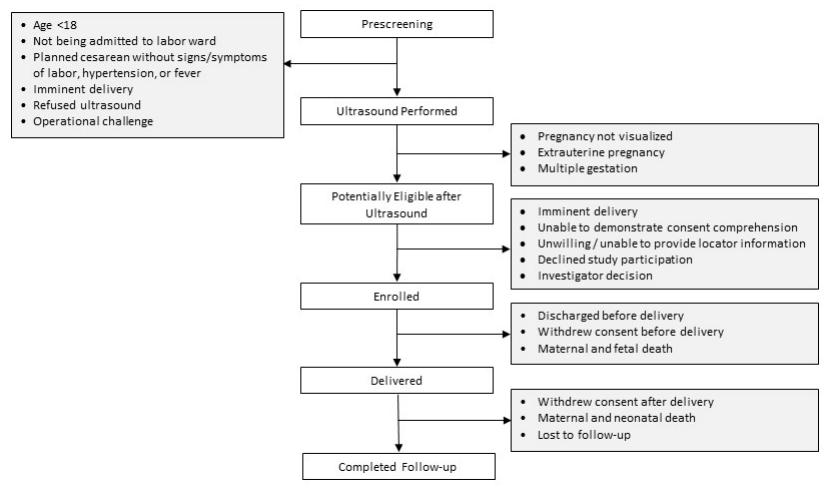
LABOR Study participant flowchart.

Women are screened for study participation at the time of presentation to the hospital for delivery and followed for 42 days after delivery. The study’s co-primary outcomes are composites of incident maternal and fetal or neonatal adverse events that may be attributed to the intrapartum period. For the mother, the primary composite outcome includes death, hemorrhage, hypertensive disorders, or infection. For the fetus/newborn the primary composite outcome includes intrapartum stillbirth, neonatal death, encephalopathy, or sepsis. The LABOR Study has been designed following the guidelines outlined by the Strengthening the Reporting of Observational Studies in Epidemiology (STROBE) statement ( Figure 1) ^7^.

The study includes a pilot phase of the first 500 mother-infant pairs enrolled at the Zambia site prior to full implementation at all sites. The primary objective of the pilot is to gain experience with study procedures and outcomes measurement, and apply lessons learned about optimal strategies for enhancing uptake, retention, data quality, and data ascertainment to the larger observational cohort.

Study execution is expected to take 5 years, including start-up and staff training (9 months), enrollment and delivery (up to 3 years), completion of follow-up (2 months), data cleaning (6 months), analysis (6 months), and dissemination of results (3 months).

### Study setting

The LABOR Study is conducted on the inpatient antepartum, labor and delivery, and postpartum wards at four sites across three countries: Korle-Bu Teaching Hospital (KBTH) in Accra, Ghana; Fakir Mohan Medical College and Hospital (FMMCH) in Balasore, India; Jawaharlal Nehru Medical College (JNMC) in Belgaum, India; and Women and Newborn Hospital of the University Teaching Hospitals (WNH-UTH) in Lusaka, Zambia. The sites were chosen based on the following criteria: large volume of deliveries, high incidence of adverse birth outcomes, and demonstrated capacity to implement rigorous clinical research.

### Study participants

Women seeking care at a study facility who meet the following inclusion criteria are eligible for participation: 1) maternal age of majority (18 years at all study sites); 2) intrauterine singleton pregnancy; 3) admission to hospital labor and delivery unit with plan for either (a) vaginal delivery or (b) cesarean delivery in the presence of signs/symptoms of parturition, elevated blood pressure, or fever; 4) willing and able to provide written informed consent; 5) willing to adhere to study procedures and provide locator information for follow-up. Women being admitted to the labor and delivery unit with a plan for cesarean delivery who have no signs or symptoms of parturition, no elevated blood pressure, and no fever are therefore excluded. We exclude women who have any condition (social or medical) that, in the opinion of study staff, would make participation unsafe or not feasible. All potential participants are assessed for eligibility and enrolled following provision of informed consent. Participants are then followed through labor, delivery, and discharge and at seven- and 42-day follow-up contacts (phone calls or in-person visits). Liveborn infants are enrolled at birth and followed through 42 days of life.

### Study procedures

***Sensitization***

Each study site conducts routine “sensitization” activities to educate community members and potential participants about the LABOR Study. Study staff meet with key stakeholders and hospital staff regularly to ensure smooth integration of study activities into provision of standard care at each study facility. Study staff also periodically provide health talks and written informational materials to women attending antenatal clinics in the catchment area of the study facilities so that potential participants are informed about the study prior to presentation for labor and delivery.

***Prescreening***

To be able to compare women who enroll in the study with those who do not, staff record basic de-identified information about all women presenting for labor at participating sites, including maternal age, gravidity, parity, estimated gestational age, presenting symptoms, and any reasons for ineligibility for enrollment. Following prescreening, all women undergo an ultrasound performed by a certified sonographer or consultant obstetrician as part of standard care prior to study enrollment. The sonographer performs a general survey and fetal biometry measurements of biparietal diameter, head circumference, abdominal circumference, femur length, and transcerebellar diameter. While sonographers are encouraged to measure and record each structure twice, if not feasible due to advanced labor, descent in the maternal pelvis, or fetal cranial calcification, the minimum required biometry element is a single femur length measurement. Based on individual biometry measurements, the estimated fetal weight, due date, and gestational age as calculated by the ultrasound machine are documented in real time. Study sonographers then perform umbilical artery doppler velocimetry on all potential participants. The entire ultrasound examination, including abdominal sweeps collected as part of the general fetal survey, is electronically archived.

***Screening and enrollment***

Following prescreening assessment of basic eligibility criteria (e.g., maternal age, singleton pregnancy, admission to labor ward, interest in study participation), potentially eligible women provide written informed consent in their preferred language. Before signing, a participant must demonstrate that she fully understands the information in the informed consent form by successfully completing a comprehension assessment. Participants provide separate consent for long-term storage of biological samples (detailed below) for possible future research testing not defined in the LABOR Study protocol. Participants may choose not to have these specimens stored for possible future research testing or withdraw their consent for long-term specimen storage at any time and remain in the study.

Study nurses collect information from consented participants about relevant maternal demographics, health and risk behaviors, and medical and obstetrical history in fixed case report forms. Research staff review the participant’s medical records to abstract relevant antenatal care, medical comorbidities, prior ultrasound findings, and key laboratory results performed during pregnancy and in the hospital prior to study enrollment. LABOR Study staff perform a physical examination and collect peripheral blood, urine, and vaginal/rectal swabs for real-time testing and storage ( Table 1).

**Table 1.** Schedule of evaluations.

	Enrollment	Labor	Delivery	Before discharge	7 days after delivery	42 days after delivery
Informed consent	•					
Collection / review of locator information	•			•	•	•
Medical chart abstraction	•	•	•	•		
Demographic / behavioral assessment	•					
Maternal physical exam	•					
Ultrasound	•					
Maternal / fetal sensor placement	•					
Labor progress assessments		•				
Maternal / fetal sensor removal				•		
Maternal and fetal / neonatal outcomes assessment		•	•	•	•	•
Neonatal physical exam				•		
Maternal point-of-care tests (HIV, syphilis, hemoglobin, urinalysis)	•					
Maternal chemistry	•					
Maternal hematology	•			•		
Maternal lactate	•					
Cord blood lactate			•			
Neonatal blood culture (if NICU admission)				•		
Maternal vaginal / rectal swabs storage	•					
Maternal blood storage	•			•		
Maternal urine storage	•					
Cord blood storage			•			
Neonatal DBS card storage				•		
Neonatal urine storage (if moderate or severe encephalopathy)				•		

NICU, neonatal intensive care unit; DBS, dried blood spot

***Sensor placement***

At enrollment, LABOR Study staff place physiologic sensors on each participant to collect maternal vital signs, maternal body position, fetal electrocardiogram, and uterine electromyogram. These data are not made available to providers for clinical care but are stored electronically for future correlation with adverse pregnancy outcomes. Continuous maternal vital signs are recorded by Sibel Health’s ANNE™ System that includes two skin-mounted sensors placed on the maternal chest and finger. Fetal ECG signals are collected continuously by the MindChild Meridian M110 Fetal Monitoring System, an intrapartum fetal monitor that uses surface electrodes embedded in four patches placed on the maternal abdomen to detect fetal and maternal ECG signals and uterine muscle contraction signals ^8, 9^. In early 2022, we transitioned to using Sibel Health’s ANNE™ Emvryo fetal ECG sensor comprising 18 external electrodes placed on the maternal abdomen ^10^. All ANNE™ devices include Bluetooth Low Energy capabilities and lithium-polymer batteries to allow use without a wired connection or external, steady electrical source.

***Intrapartum procedures***

Following enrollment, LABOR Study staff continuously collect data through labor, delivery, and immediately after delivery. To accomplish this real-time task, we introduced study “scribes,” a cadre of data collection specialists tasked with collecting detailed information by direct observation, interrogation of clinical staff, and medical record abstraction. The scribes have no clinical role; on-site clinical care providers make all decisions and diagnoses regarding labor management in accordance with practice prevailing at each study site.

Scribes are positioned at participants’ bedsides and enter data in real time into a tablet-based data management system, built using Dimagi’s CommCare platform ^11^ and customized for use in the LABOR Study. Scribes are assigned up to three participants simultaneously, depending on volume and acuity of active intrapartum participants. Although real-time data collection is encouraged, when multiple events occur simultaneously or the scribe needs time to confirm observed events, data may be entered retrospectively with a back-dated timestamp.

Throughout labor, delivery, and immediately postpartum, the scribes are responsible for recording detailed information about all actions performed, medications provided, and diagnoses made for each participant. Scribes document all actions carried out either by clinical staff or the participant herself, which may include assessments (e.g., cervical examination), procedures (e.g., amniotomy), or events (e.g., maternal pushing). Information recorded about medications administered includes name, route of administration, dose, and time administered. Scribes document all diagnoses made by on-site clinical care providers throughout labor, delivery, and immediately postpartum. Finally, they are responsible for ensuring continuous data collection from all study sensors and documenting any adjustments or interruptions.

During delivery and immediately postpartum, scribes are encouraged to prioritize real-time documentation of time and mode of delivery, time of cord clamping and placental delivery, estimated blood loss measured by a study-supplied calibrated drape (for vaginal deliveries), and any delivery complications. If scribes are present for cesarean delivery, they are additionally responsible for documenting the time and type of skin and uterine incisions. After delivery, scribes document neonatal vital status, Apgar scores, sex, birth weight, head circumference, and provision of any positive pressure ventilation.

Immediately after the umbilical cord is clamped and cut, a sample of umbilical venous blood is drawn and tested for lactate concentration on a point-of-care platform. Additional venous cord blood is also processed and stored for future testing.

***Postpartum maternal and neonatal procedures***

After transfer from the labor ward, LABOR Study staff closely track mother-infant pairs, visit each participant at least daily, and perform abstraction of maternal and neonatal medical charts. Study staff document maternal postpartum care, neonatal care, and final maternal and neonatal outcomes prior to hospital discharge. An additional maternal blood sample is collected once the mother is stabilized following transfer from the delivery room for immediate hematology analysis and storage for future testing.

Trained and certified study providers perform up to two neurological examinations to diagnose intrapartum-related neonatal encephalopathy for newborns weighing at least 1500 grams who meet any one of the following four physiologic criteria: ten-minute Apgar score <5, continued need for positive pressure ventilation at ten minutes of life, venous cord blood lactate >7mmol/L, or acute perinatal event, defined as intrapartum hemorrhage, umbilical cord prolapse, fetal distress as evidenced by an abnormal heart rate or pattern, or uterine rupture. The newborn neurological examination is based the modified Sarnat examination that evaluates level of consciousness, spontaneous movement, posture, tone, primitive reflexes, and the autonomic nervous system ^12, 13^. For all newborns diagnosed with either moderate or severe encephalopathy by neurological examination, urine specimens are collected and stored for future testing. One urine specimen is collected on the second day of life and another on day seven of life or discharge, whichever occurs first.

All newborns admitted to the neonatal intensive care unit within the first seven days of life are assessed for signs or symptoms of sepsis, and a blood culture is obtained (prior to antibiotics, if administered). Repeat blood cultures may be collected in cases of new, worsening, or recurrent signs or symptoms of sepsis during the first seven days of life. LABOR Study staff collect a dried blood spot card on all study newborns at 24 hours after delivery or discharge, whichever occurs first, to be stored for future analyses.

At discharge, LABOR Study staff complete a final review of the maternal and neonatal hospital course and reconcile key study diagnoses, medications, laboratory and radiographic diagnostic tests, and other interventions that occurred during labor, delivery, and the inpatient postpartum period. Study staff are responsible for confirming or correcting data recorded by scribes during the intrapartum and immediate postpartum periods and for adding any additional diagnoses and/or interventions that have occurred since delivery as documented in the medical record or in study chart notes. Finally, study staff categorize birth outcomes according to a modified rubric to assign a clinical delivery phenotype ^14^.

Approximately seven and 42 days after delivery, LABOR Study staff contact participants by phone (or in person as needed) to assess maternal and neonatal status and interval course. Staff collect information about maternal and neonatal vital status and events since discharge, including signs and symptoms experienced, non-routine clinic or hospital visits attended, diagnoses made, and treatments received.

### Laboratory procedures

All study samples are obtained from study participants by trained study staff according to approved standard operating procedures. Specimen transport, processing, storage, testing, and results reporting are conducted in accordance with Good Clinical Laboratory Practice (GCLP) standards ^15^. Samples are processed according to the assay manufacturers’ specifications by certified laboratory personnel. All relevant materials, including diagnostic specimens and infectious substances, are transported according to instructions detailed in the International Air Transport Association (IATA) Dangerous Goods Regulations. Potentially biohazardous waste is managed according to institutional, national, and other applicable regulations.

Whenever possible, maternal and neonatal blood samples are taken at the time of regularly scheduled clinical blood draws. Specimens are analyzed immediately as described in Table 1 and stored at -80°C for later study-related analysis to identify inflammatory markers, metabolic analytes, proteins, hormones, transcripts, and infectious factors contributing to adverse birth outcomes.

### Data management

**Figure 2.**
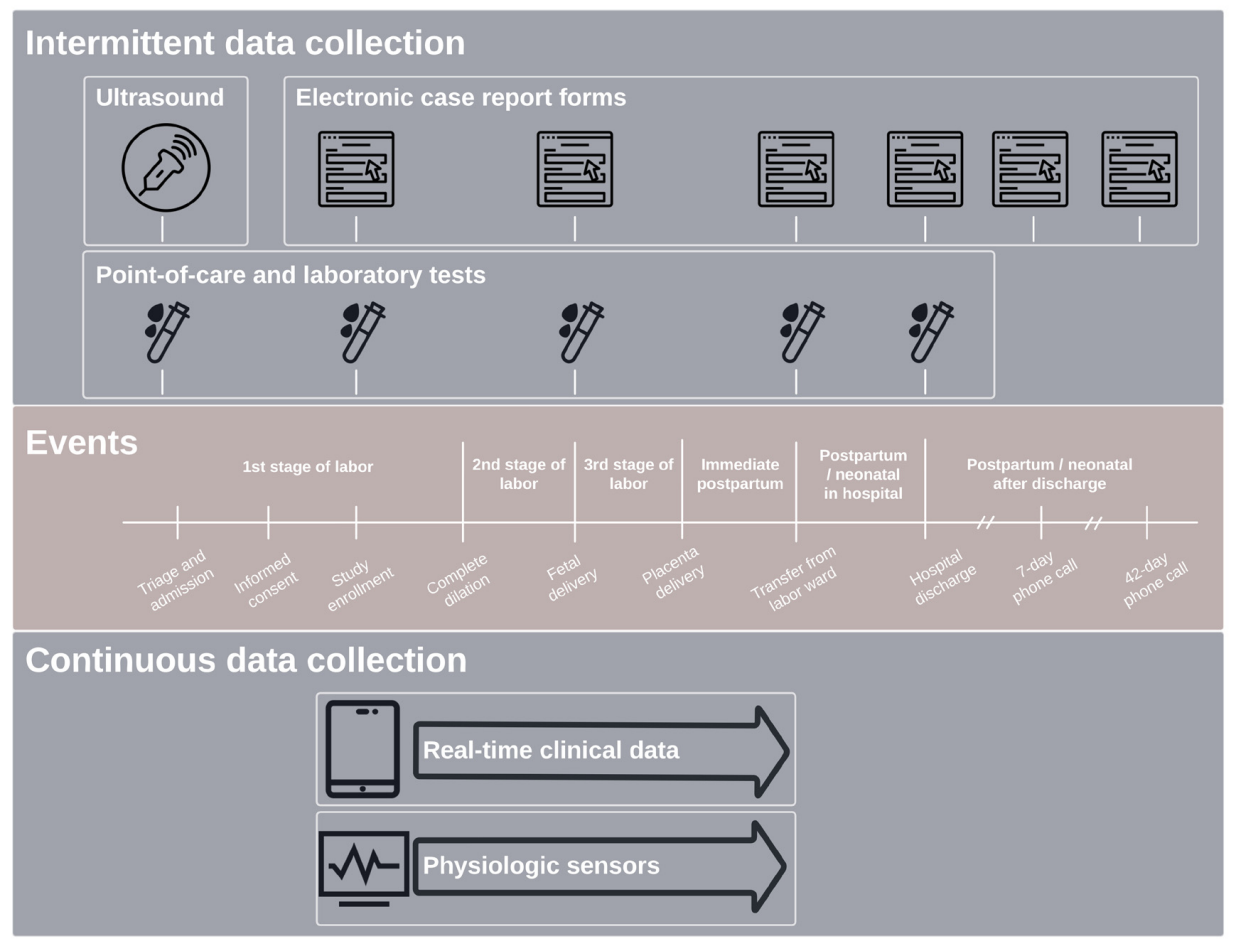
LABOR Study data collection.

The study database has four primary sources of data as follows ( Figure 2): (1) fixed case report forms and real-time scribe data, managed via the CommCare system; (2) laboratory data, managed via CommCare and Lab Data Management Systems; (3) fetal and maternal physiologic sensor data, managed via a local server synchronized with cloud-based servers; and (4) ultrasound data, managed via a cloud-based server (Trice Imaging Inc, Del Mar, CA, USA). Data from each of these sources are automatically transferred to a secure, centralized server where they are queried, curated, and cleaned as needed. Key information from these sources is processed, synthesized, and presented on site-specific, custom-built R Shiny dashboards that are hosted on a secure, self-managed Shiny server and deployed on the web. Using data directly from the centralized server, dashboard metrics and visualizations are computed on demand without any required intermediate hand processing and available to authorized study staff at any time. Access to real-time study metrics allows the study operating center and site staff to track performance and identify challenges as they arise. 

### Data analysis and statistical plan

***Study outcomes***

The primary outcomes of the LABOR Study include a composite maternal outcome of potentially actionable incident adverse events and a composite fetal / neonatal outcome of the same nature ( Table 2). Secondary outcomes include the individual components of the two primary composite outcomes: maternal outcomes including maternal death, incident intrapartum hemorrhage, postpartum hemorrhage, incident hypertensive disorders (eclampsia, severe preeclampsia, preeclampsia, superimposed preeclampsia, severe gestational hypertension), and incident maternal infection (intrauterine inflammation or infection, endometritis), and fetal / neonatal outcomes including intrapartum stillbirth, neonatal death, encephalopathy, and sepsis. The LABOR Study distinguishes between prevalent conditions (those present at the time of enrollment) and incident conditions (those arising after enrollment). Although we identify and assess frequency of prevalent conditions, the primary focus of analytic approaches described below is the characterization and prediction of incident conditions.

**Table 2.** Study outcome definitions.

Maternal Outcomes	
Maternal death	Death of a maternal participant after study enrollment and up to 42 days postpartum, irrespective of cause
Incident intrapartum hemorrhage	Intrapartum vaginal bleeding of ≥ 1000 mL of blood prior to delivery of the placenta
Postpartum hemorrhage	Blood loss of ≥ 1000 mL or any bleeding leading to an adverse event, regardless of mode of delivery, either within the 24 hours after delivery of the placenta (primary) or up to 42 days postpartum (secondary)
Incident eclampsia	New onset of tonic-clonic, focal, or multi-focal seizures in the absence of other causative conditions
Incident severe preeclampsia	New development of systolic blood pressure ≥ 140 mmHg or diastolic pressure ≥ 90 mmHg measured at least 4 hours apart in addition to proteinuria and at least one of the following severe features: thrombocytopenia, renal insufficiency, impaired liver function, pulmonary edema, new-onset cerebral or visual disturbances, or rise in systolic blood pressure to ≥ 160 mmHg or diastolic pressure to ≥ 110 mmHg
Incident preeclampsia	New development of systolic blood pressure ≥140 mmHg or diastolic pressure ≥ 90 mmHg measured at least 4 hours apart in addition to proteinuria in the absence of severe features described above
Incident superimposed preeclampsia	New development of preeclampsia as defined above in a woman with chronic hypertension
Incident severe gestational hypertension	New development of severe range blood pressure (i.e., ≥ 160 mmHg systolic pressure or ≥ 110 mmHg diastolic pressure) without proteinuria, thrombocytopenia, renal insufficiency, impaired liver function, pulmonary edema, or cerebral or visual disturbances
Incident intrauterine inflammation or infection	Maternal fever and any of the following prior to delivery: fetal tachycardia, maternal leukocytosis, drainage of purulent fluid from cervical os
Endometritis	Presence of two or more of the following on any 2 of the first 10 days postpartum, exclusive of the first 24 hours and with no other recognized cause: maternal fever, abdominal pain, uterine tenderness, purulent drainage from uterus
Fetal / Neonatal Outcomes	
Intrapartum stillbirth	A stillborn fetus weighing at least 500g meeting all of the following criteria at birth: no heart rate, no respirations, absence of pulsation of the umbilical cord, and no definitive movement of voluntary muscles, and in which fetal heart tones were documented on the ultrasound performed at the time of LABOR Study enrollment
Neonatal death	Death of a live-born neonate within 28 days of birth
Neonatal encephalopathy	Live-born neonate weighing at least 1500g that meets one of the following four physiologic criteria: venous cord lactate > 7 mmol/L; 10 minute APGAR < 5; continued need for ventilation at ≥ 10 minutes of life; intrapartum hemorrhage, cord prolapse, severe fetal heart rate abnormality, or uterine rupture, and that demonstrates moderately or severely abnormal neurologic findings in at least three of the following six categories: spontaneous activity, posture, level of consciousness, tone, primitive reflexes, autonomic nervous system.
Neonatal sepsis	Live-born neonate exhibiting one or more signs of early-onset sepsis (i.e., convulsions, tachypnea, severe chest indrawing, nasal flaring, grunting, bulging fontanelle, pus draining from ear, umbilical redness extending to the skin, fever, low body temperature, many or severe skin pustules, lethargy / unconsciousness, or less than normal movement) with a positive blood culture (excluding contaminants) within the first seven days of life

***Sample size***

Because the incidence rates of certain complications of interest are small, we require a large sample size to observe sufficient numbers of each complication. During initial study planning, we calculated a sample size of 15,000 participants across three countries (four sites), with enrollment evenly distributed among the countries. However, with start-up and accrual delays owing to the SARS-CoV2 pandemic, we conducted a modified analysis to assess the impact of a reduced sample size of 12,000 on our key analytic goals. To do this, we performed a 2-part simulation study to assess our expected ability to (1) estimate risk and (2) estimate optimal decision rules. For (1), we assessed the anticipated precision with which we would be able to estimate risk for key outcomes using exact Poisson confidence intervals ^16^. Basing our estimates on observed event rates in the first 500 participants in the study, we expect to estimate a rare event, one occurring in just 2% of the participants, with 95% confidence intervals with a half-width of 1.2%. For (2), using the first 500 observations in the study and 9 key covariates, we generated simulation data sets by sampling with replacement from the observed covariates and constructing binary treatment decisions for each simulation record conditional on covariates. We also generated outcomes for each simulation record such that individual treatment effects exhibited heterogeneity dependent on interactions between the treatment and patient covariates. For each simulation data set, we estimated an optimal treatment rule that tailors treatment recommendations based on patient features. Across replications, we estimated the probability of correctly rejecting the null hypothesis that there is no benefit of personalized decision-making when compared to completely randomized decision-making (i.e., a coin flip). With a true risk ratio of 1.17 and a sample size of 12,000 participants, our analysis indicates a 76% probability of correctly rejecting the null hypothesis. Our simulation results further indicate that an overall sample size of 12,000 yields sufficient power for estimating decision rules that, when used to recommend treatments, will reduce the probability of observing a bad outcome sufficiently close to the true optimal.

***Statistical analysis***

*Descriptive analysis of baseline, intrapartum, and outcome variables.* Common methods for descriptive statistics will be used to examine the distribution of key baseline factors, intrapartum events, and overall outcomes in the study population (e.g., frequencies for categorical variables and measures of centrality and dispersion for quantitative variables). These methods will also be used to examine unadjusted (crude) differences in the distribution of outcomes and intrapartum events by baseline factors. Key baseline factors will include maternal demographics, medical history and obstetrical risk factors, enrollment ultrasound, physical examination and laboratory values collected at admission or enrollment in the study. Intrapartum events will include time-varying variables related to the course of labor (e.g., cervical examinations), provider interventions (e.g., drugs, procedures), and continuous monitoring through fetal and maternal sensors.

*Associations between baseline variables, interventions, and outcomes.* Generalized linear models will be used to estimate the adjusted relative risk with 95% confidence interval of outcomes associated with baseline factors. Covariates (baseline factors) for inclusion in the multivariable models will be identified *a priori* based on clinical relevance and using causal directed acyclic graphs. We will start by fitting two separate models for binary outcome variables (yes/no) for the composite maternal and fetal / neonatal outcomes, respectively. If baseline factors of interest are found to be associated with the composite outcomes, we will fit models to each of the individual outcome components within each composite (maternal or fetal / neonatal). A similar modeling approach to that used for the composite and individual outcomes will be used to estimate associations between baseline factors (covariates) and key intrapartum interventions such as cesarean delivery.

*Predictive models and risk stratification.* We will develop and validate models to use baseline variables to predict the probability of each outcome. These predicted probabilities can be used to stratify patients by risk. Because of the expected high dimensionality of the data, we will consider the use of dimension reduction techniques such as deep learning autoencoders ^17^, a nonlinear feature extraction tool using artificial neural networks, to reduce the dimension of baseline data prior to modeling, as well as, when appropriate, inclusion of regularization techniques such as elastic net ^18^. Predictive models will be fit using multiple machine learning techniques such as random forests ^19^, reinforcement learning trees ^20^, and kernel support vector machine ^21^. Model selection and validation will be done within a cross-validation framework with models fit using a training set, hyperparameters tuned using a validation set, and performance assessed on a testing set. For binary outcomes, model fit may be assessed using metrics such as accuracy, and for continuous outcomes, model fit may be assessed using metrics such as root mean squared error (RMSE). Key features identified in the predictive modeling will help us identify potential tailoring variables to be included in subsequent data-driven clinical decision support algorithms.

*Signal processing and dimension reduction.* The expected availability of multiple streams of physiological signals including maternal heart rate, contractions, and fetal heart rate in risk prediction models and clinical decision support algorithms is a unique opportunity. Yet, given the differences in the volume and velocity between these high-dimensional signal data and more standard clinical examination and laboratory data elements, some signal processing including possibly dimension reduction and feature extraction will be needed before the various types of data can be integrated into statistical learning models. A number of signal processing strategies are available; for example, Fourier decomposition methods may be used to analyze the nonlinear and non-stationary time series ^22^, the aforementioned autoencoder ^23^ may be used to reduce signal dimensionality to a parsimonious class of features, and modern deep learning may be used on the raw signal directly to extract and construct novel, highly predictive features.

*Finite horizon and infinite horizon dynamic treatment regimes.* Beyond risk prediction, modern statistical precision medicine methods will be used to generate optimal, data-driven treatment recommendations. The statistical precision medicine framework formalizes the clinical decision-making process into a statistical model with baseline and time-evolving patient features, a sequence of key decision points, a set of feasible treatment options available at each decision point, and an outcome to optimize (i.e., either the maximization of a positive outcome or the minimization of a negative outcome). Framed within a rigorous causal framework, the goal is to learn from the data optimal dynamic treatment regimes (DTRs), which are sometimes referred to as individualized treatment regimes (ITRs). DTRs are a sequence of decision rules, one for each key decision point, that yield an optimal treatment recommendation given an individual patient’s health status and history up to that decision point; they may take a multitude of forms, ranging from interpretable if-else decision lists to black box algorithms. Optimal DTRs are DTRs that, if followed, would improve outcomes on average across the population ^24^. Distinct from risk prediction, learning optimal DTRs is focused on improving decision-making and tailoring decisions to account for inter-patient heterogeneity.

We will assess the feasibility of analyzing a key decision or sequence of decisions using formal causal inference tools, including causal directed acyclic graphs and assessments of whether there are structural or practical positivity violations. If feasible, we will model key labor decisions in a Markov decision process (MDP) framework ^25^. For a given decision problem or sequence of related decisions, we will first analyze decision-making using a *finite-time horizon* where decision time points are fixed, yielding one decision rule for each decision point that optimizes a targeted outcome across all of the decision recommendations. Optimal DTRs in this setting will be learned via off-policy, finite-time horizon reinforcement learning methods such as Q-learning ^26^ or simultaneous outcome weighted learning ^27^. When warranted by the decision problem at hand, we may then estimate an optimal DTR within an *infinite horizon* framework, where the decisions can be made recurrently with virtually no limit on the number of decision times. In this case, the estimated treatment regime provides real-time recommended actions based on both baseline and time-varying information rather than at pre-specified time points and is focused primarily on optimizing imminent outcomes. We will utilize reinforcement learning methods developed for infinite horizon problems such as V-learning ^28^ and greedy gradient Q-learning ^29^.

*Missing data.* As part of ongoing data quality assurance and control procedures, we monitor and promote high completion of key study variables before the end of follow-up, including baseline variables, intrapartum interventions, and variables used to define outcomes. In the analysis phase, we will investigate patterns of missingness and consider the use of multiple imputation or weighted estimation methods, as appropriate, to account for missing data when the proportion of participants missing data is more than 20% ^30^. We will also report complete case analyses alongside multiple imputation or weighted estimation to assess the impact of missing data.

### Ethical considerations

The study is conducted according to international standards of Good Clinical Practice ^31^. The protocol and informed consent documents are approved by properly constituted independent Ethics Committees (ECs) or Institutional Review Boards (IRBs) maintaining Federal Wide Assurances (FWA) with the Office for Human Research Protections (OHRP) at each site. All staff with participant contact receive training on the protection of human research participants prior to conducting any study activities and routinely thereafter ^32^.

Participation in the study is voluntary. All study participants are provided a consent form describing the study and providing sufficient information for participants to make an informed decision about their participation in the study. LABOR Study consent forms are translated into local languages and back-translated into English to ensure accurate translation. Potential participants have the choice of undergoing consent in English (all sites); Hindi, Kannada, or Marathi (Belgaum, India); Bengali, Hindi, or Oriya (Balasore, India); Ewe, Ga, or Twi (Ghana); or Nyanja or Bemba (Zambia). Clinical study procedures are conducted according to local standards of routine care. Study staff inform women that they do not have to agree to participate in the study to receive care at the delivery facility even if investigators are affiliated with both the facility and the study.

Investigators make every reasonable effort to minimize risks to participants. We expect that participants in this observational study will be exposed to minimal risk. Physical risks of participation include risks associated with venipuncture or finger prick, which are infrequent and minimized with use of proper technique. Collection of vaginal and rectal samples may also be associated with some discomfort. To reduce the risk of social or psychological harm, we explain the study in detail during the informed consent process and routinely remind participants that they do not have to answer questions that make them uncomfortable. To maintain participant confidentiality, all research-related discussions and informed consent procedures are conducted in a private room or area. All study data, laboratory specimens, and administrative forms are identified by a unique coded number. This unique number is scanned anytime data are captured within the password-protected study tablets. All databases are password-protected and accessible only to members of the study team. 

The study has been approved by the following committees: University of North Carolina IRB, Chapel Hill, NC, USA; University of Ghana College of Health Sciences Ethical and Protocol Review Committee, Accra, Ghana; Korle Bu Teaching Hospital IRB, Accra, Ghana; Institutional Ethics Committee of KLE Academy of Higher Education and Research, Belgaum, Karnataka, India; Institutional Ethics Committee of Fakir Mohan Medical College and Hospital Balasore, Odisha, India; India Research and Ethics Committee, Directorate of Health Services, Odisha, India; Health Ministry Screening Committee of Government of India with Indian Council of Medical Research acting as its Secretariat; University of Zambia Biomedical Research Ethics Committee, Lusaka, Zambia; Zambia National Health Research Authority, Lusaka, Zambia.

### Safety monitoring

At each study contact, study staff evaluate participants for social harms and adverse events. All adverse events occurring after study enrollment are assessed for seriousness and relatedness to study procedures. Study staff document and carefully monitor all serious adverse events occurring in participants whether or not they are related to study participation. The severity of any serious adverse events is graded using the National Institute of Health’s Division of AIDS Table for Grading the Severity of Adult and Pediatric Adverse Events. Study staff are responsible for promptly reporting related adverse events or social harms to the site investigators and the overall study Principal Investigator, who determine if reporting to the appropriate ethics committees is required based on their guidelines. 

### Dissemination of findings

The LABOR Study team will disseminate findings to participants and key stakeholders at local dissemination events and globally at international conferences and in peer-reviewed journals. At the completion of this study, in accordance with the Bill and Melinda Gates Foundation Global Access requirements, research data from participants followed through pregnancy and the postpartum period will be curated and documented, stripped of personal health information, and placed into the public domain. Additionally, the study team welcomes collaboration with others who could make use of intrapartum testing algorithms developed upon study completion. Knowledge generated from this study has the potential to contribute to the development of a suite of tools (which may include diagnostics, devices, and/or software) that can be deployed in low-resource healthcare facilities to substantially improve intrapartum outcomes in low-resource settings worldwide.

### Study leadership and oversight

The LABOR Study is funded by the Bill and Melinda Gates Foundation and sponsored/coordinated by the University of North Carolina at Chapel Hill. The Management Team provides central technical and operations expertise to sites to ensure the successful implementation of the study and is responsible for developing the clinical protocol, training study staff, monitoring recruitment and retention, managing data quality, and ensuring regulatory compliance. The Steering Committee is led by the study Principal Investigator and comprises representatives from each participating site and additional ad hoc members selected for domain-specific knowledge. The Steering Committee advises on overall operations and manages all decisions concerning data analysis and interpretation, secondary analyses, and dissemination.

### Study status

We completed participant follow-up at all sites in September 2022.

## Discussion

Provision of quality obstetrical and neonatal health care is a complex matter that requires informed patients, trained providers, equipped and proximal facilities, timely diagnostics, an uninterrupted supply of essential treatments, and a coordinated strategy for all individual components to operate harmoniously ^33^. Obstetrical and neonatal services in resource-limited settings often lack some or all of these critical components. Algorithms that identify the maternal-fetal dyads at greatest risk for preventable intrapartum complications would allow the targeting of limited resources toward those that need it most and are the most likely to benefit.

The overarching goal of our research is to develop new tools that can be deployed in low-resource healthcare facilities to improve intrapartum outcomes through better risk stratification, timely intervention or transfer to higher care, and earlier diagnosis. The first step toward this goal is to precisely characterize the incidence of adverse labor outcomes and their measurable antecedents in diverse populations while carefully documenting patterns of care. The data generated by this study will serve as a foundation for new discovery and development of novel technological approaches to substantially reduce adverse perinatal outcomes. With this study, we aim to create the largest, most extensively documented observational cohort of labor and delivery ever assembled.

## Data availability

No data are associated with this article.
