# Supplementary material for: Limiting adverse birth outcomes in resource-limited settings (LABOR): protocol of a prospective intrapartum cohort study
Source: Gates Open Res. 2022 Dec 19;6:115. Originally published 2022 Aug 24. [Version 2] doi: 10.12688/gatesopenres.13716.2 (PMC9822935; doi:10.12688/gatesopenres.13716.2)
Supplement: Peer review history

# Peer review: Reviewer response for version 2

**DOI:** 10.21956/gatesopenres.15569.r32809

**Published:** 2023-01-06

**Authors:** Soo Downe

**Affiliations:** 1 THRIVE Centre, School of Community Health and Midwifery, University of Central Lancashire, Preston, UK

Thank you for the detailed responses to my comments. I am reassured that the findings of the study will be subject to extensive translation into clinical practice through trials and implementation science approaches, especially to confirm the feasibility, affordability, equity and effectiveness of using the proposed approach to personalised risk assessment. I am also reassured that some of the limitations addressed in both reviewers' comments, and acknowledged in the response will be addressed in the publication of the results of the study. On this basis, I am happy to approve the report.

Is the study design appropriate for the research question?

Partly

Is the rationale for, and objectives of, the study clearly described?

Yes

Are sufficient details of the methods provided to allow replication by others?

Yes

Are the datasets clearly presented in a useable and accessible format?

Not applicable

Reviewer Expertise:

Relevant to this proposal: Maternity care organisation and practice; Health systems research; Quality of maternity care

I confirm that I have read this submission and believe that I have an appropriate level of expertise to confirm that it is of an acceptable scientific standard.

# Peer review: Reviewer response for version 1

**DOI:** 10.21956/gatesopenres.15003.r32414

**Published:** 2022-10-17

**Authors:** Soo Downe

**Affiliations:** 1 THRIVE Centre, School of Community Health and Midwifery, University of Central Lancashire, Preston, UK

This is a protocol for a study that appears to be complete, based on the statement by the authors under the heading 'study status' that they expect to complete enrolment and follow up by the second quarter of 2022. 

**Overview**

 The protocol sets out plans for an ambitious and complex study to be run in multiple countries. It aims to identify maternal-fetal dyads at greatest risk of preventable intrapartum complications, to allow targetting of resources, with the hypothesis that this will improve outcomes for those most likely to benefit.

**Comment**

 The ambition, reach, and attention to detail displayed in the protocol are highly laudable. Planned processes and procedures are clearly explained, and the addition of scribes in the labour ward is likely to address some of the problems of completeness and accuracy that other studies have encountered when trying to use routinely collected data (paper-based or online) for this kind of study. 

 I do have some caveats, and these relate to my scoring of 'partly' in relation to the fit between the research question and the study design. Most of these issues arise from the evidence that, while outcomes for mothers and babies are partly a function of their physical characteristics and interconnections, outcomes are also mediated by staff and organisational attitudes and behaviours, rules, guidelines, and procedures. For example, the most recent Cochrane review on the topic suggests routine use of EFM for low (and even high) risk women will result in higher CS rates than the use of intermittent monitoring, with no additional benefit to the baby long term ^1^. Some women labouring in organisations that routinely use EFM will therefore experience CS, and, potentially, post-surgical complications such as PPH, regardless of the intrinsic mother/baby dyad risks.

 Similarly, some women labouring in sites where there is limited staff available will experience adverse events that will be prevented in sites where the staff/woman ratio is higher. Those who have further to travel, or who are handed over between institutions with minimal transfer of information may experience worse outcomes than those with similar clinical characteristics who live closer to a facility - and so on.

 The issue here is that, if the locus of potential adverse outcome is only sited with the mother/fetus in any future algorithm for resource allocation, there may be a risk of both under or overdiagnosis, and, subsequently, both under and over-treatment - both of which could cause harm. 

 It may be that the statistical analysis will control for these organisational and human factors, which will then remove these concerns. However, even if that is the case, I was interested in this comment: 'O *ptimal DTRs are DTRs that, if followed, would improve outcomes on average across the population'.* This implies that the output from the study will not be personalised to individuals, but to a cluster of phenomena that might be associated with a certain outcome. The critical issue in practice is then how sensitive and specific the tool is for particular individuals (i.e., how personalised it actually is) and to what extent both professional experts and service users will have the freedom to offer/accept/decline treatment based on the potential risk identified, as opposed to on actual manifestation of pathology. 

 It will be critical for implementation research to be undertaken with the tool to establish its acceptability, feasibility, affordability, equity, and possible unintended consequences or opportunity costs before it is irretrievably embedded into organisational and electronic systems, and, therefore, practice.  

**Additional comments and issues**

1. I wasn’t clear if the hospitals included in the study are typical for those to which the results are to be applied in the future. Women labour differently and have different outcomes in settings where they are well supported rather than not, especially if they have continuous labour companionship throughout labour ^2^. It would be good to know if the analysis will control for the different types of setting for the data collection, to see how this factors into the patterns emerging.
2. The inclusion criteria include the ability to provide ' *written informed consent’/locator information'.* The exclusion criteria encompass those with ‘ *any social or medical condition that would make participation unsafe or not feasible’* . This does seem to have the potential to exclude women who are at most risk of the kinds of adverse outcomes of interest to the study (since being illiterate or living in dangerous social circumstances/having a severe medical condition is likely to be associated with the adverse outcome). Maternal or fetal deaths also seem to be excluded in the flow chart on p5, but they are included in the composite outcome. I’m not quite clear how this has operated in the study.  
3. I couldn’t tell from the protocol if women consented to the study antenatally or in labour. If in labour, I wasn’t sure how the extensive data collection could be explained in a way that would ensure the woman really understood what she was signing up for.
4. How accurate are the following measures when done as a one-off?: *the estimated fetal weight, due date, and gestational age as calculated by the ultrasound machine are documented in real-time*
5. The protocol seems to suggest that the woman has (four or sixteen) sensors placed to measure fetal ECG and maternal vital signs from the time of enrolment (antenatally for some women?) until they are discharged from the hospital. I wasn’t sure if there had been any consideration in the analysis of how having up to 16 sensors placed might impact on the freedom of movement of the mother in labour (and therefore on labour progress) even if they are wireless. I presume that the technology has been tested prior to the study to assess if it is unobtrusive, and robust (for example, in the exertion of labour, and the consequent rise in skin temperature/maternal sweating, do the sensors stay firmly fixed, or do staff have to keep re-siting them, interrupting the women’s concentration on managing her labour?). After birth, if the woman is breastfeeding, it would be useful to know if this impacts the sensors/signals, and how the sensors might impact the success of breastfeeding, if at all. It would be helpful if the final report provides an assessment of the acceptability and feasibility of the sensors during labour and birth and the postpartum period, especially if the proposal is that similar sensors might be used in routine practice in the future for women deemed to be high risk.
6. I note that the scribes are allocated up to three women simultaneously and that they are supposed to collect a large amount of data in real-time. I wonder if this is actually possible to do with accuracy and completeness. I imagine that this means that up to three women are labouring in one room. If this is indeed the environment in which these data are collected, this needs to be a caveat on the findings, since this kind of context may affect labour progress and outcome. It would be very helpful to have a description of the context and organisational labour room norms in the final report, to establish how far they may or may not have influenced the clinical progress and outcomes of labour and birth. This could include if women were upright and mobile or supine, or in lithotomy, if fundal pressure was used, and if the women had companionship, nutrition, pain relief, and respectful care as recommended by WHO. 
7. The primary outcome measure is a composite. While this makes sense at a population level, it doesn’t really lend itself to a determination about what to do about individual risk. The problem with composite outcomes is that the aetiology and therefore treatment of different components of the composite are very different. While the individual components are included as secondary outcomes, it isn’t clear if there will be sufficient statistical power to establish risk profiles for individuals with these specific outcomes.
8. The sample size calculation section states that the numbers anticipated could provide statistically significant findings for *‘a rare event, one occurring in just 2% of the participants’*'. Presumably then this doesn’t include maternal death. It would, however, probably be sufficient for perinatal mortality in the included countries, though, as noted above, it isn’t clear if these are exclusions for the study, despite the fact that they are included in the composite measure of outcome.

**Dissemination plan**

 This looks reasonable. It is good to see the intention to share the findings with local participants and key stakeholders, and with academics at conferences and in journals.  However, I can't see any plans for sharing with service user organisations more widely, or with national professional groups and policymakers. It might be useful to add this in to the plans going forward.

**Statistical plan**

 I am not a statistical expert, so cannot comment on this part of the protocol.

**Conclusion**

 As stated above, this is an important and ambitious study. It seems to be carefully designed, and it is very likely to produce a very large amount of useful data.

 My main concerns are, firstly, about the extent to which assumptions are made that risk is only a function of the clinical characteristics of the mother/fetal dyad, without considering personal, social, and health care systems/organisational impacts on labour progress and outcome; and, secondly, the risks of the rapid introduction of any algorithm resulting from the study without proper assessment of the benefits and harms of implementation in the real world. 

 If both of these concerns are addressed in the final report and in future studies and practice, I am sure that the findings will be of critical importance in future maternity care.

Is the study design appropriate for the research question?

Partly

Is the rationale for, and objectives of, the study clearly described?

Yes

Are sufficient details of the methods provided to allow replication by others?

Yes

Are the datasets clearly presented in a useable and accessible format?

Not applicable

Reviewer Expertise:

Relevant to this proposal: Maternity care organisation and practice; Health systems research; Quality of maternity care

I confirm that I have read this submission and believe that I have an appropriate level of expertise to confirm that it is of an acceptable scientific standard, however I have significant reservations, as outlined above.

## References

[1] : Continuous cardiotocography (CTG) as a form of electronic fetal monitoring (EFM) for fetal assessment during labour. *Cochrane Database of Systematic Reviews* .2017;2019(5) : 10.1002/14651858.CD006066.pub3 10.1002/14651858.CD006066.pub3 16856111

[2] : Continuous support for women during childbirth. *Cochrane Database of Systematic Reviews* .2017;2017(8) : 10.1002/14651858.CD003766.pub6 10.1002/14651858.CD003766.pub6 PMC648312328681500

# Response

**Published:** 2022-12-05

**Authors:** Joan Price

We greatly appreciate your careful review and commentary. Please find point-by-point responses to your questions and comments below.

**I wasn’t clear if the hospitals included in the study are typical for those to which the results are to be applied in the future. Women labour differently and have different outcomes in settings where they are well supported rather than not, especially if they have continuous labour companionship throughout labour. It would be good to know if the analysis will control for the different types of setting for the data collection, to see how this factors into the patterns emerging.**

 Each of the 4 study sites are hospitals based in low-resource urban areas. WNH-UTH, KBTH, and JNMC are tertiary teaching hospitals. FMMCH in Balasore is a busy second-level public hospital. We did not collect information about labor companionship since the WHO guidance for encouraging a companion had not been universally implemented at the time of the study. We do plan to explore other differences in site data that may have been impacted by setting (see concluding paragraphs below).

**The inclusion criteria include the ability to provide ' *written informed consent’/locator information'.* The exclusion criteria encompass those with ‘ *any social or medical condition that would make participation unsafe or not feasible’* . This does seem to have the potential to exclude women who are at most risk of the kinds of adverse outcomes of interest to the study (since being illiterate or living in dangerous social circumstances/having a severe medical condition is likely to be associated with the adverse outcome). Maternal or fetal deaths also seem to be excluded in the flow chart on p5, but they are included in the composite outcome. I’m not quite clear how this has operated in the study.**

 Requiring written informed consent introduces a limitation to the generalization of our dataset, a drawback that we will acknowledge when reporting results of the study. We attempt to approach all women presenting to the delivery room at each study site and offer them enrollment in the study based on preliminary eligibility (i.e., age, intent to deliver vaginally, singleton pregnancy) and interest in study participation. The most common reason that women are not able to provide informed consent is imminent delivery. Study staff were trained to identify individuals to serve as impartial witnesses during the consent process for any illiterate participants. Women with severe medical conditions are eligible to participate provided their condition did not interfere with the ability to provide consent (e.g., they were mentally disabled, unconscious, or receiving intensive care prior to delivery). The requirement to provide locator information is a telephone number only to allow for follow-up phone calls; participants were not required to provide an address.

 Maternal or fetal deaths occurring after enrollment will be analyzed as study outcomes. We collect detailed information on all participants who died up to the time of death of both members of the mother-infant pair, who are then excluded from further follow-up activities.

**I couldn’t tell from the protocol if women consented to the study antenatally or in labour. If in labour, I wasn’t sure how the extensive data collection could be explained in a way that would ensure the woman really understood what she was signing up for.**

 Most women consent while in labor. As stated above, some that are approached are too advanced for study consent to be feasible. To enhance comprehension of the consent form upon admission for labor and delivery, research staff routinely discuss the study with women receiving antenatal care who are likely to deliver at the study facilities. All participants are required to demonstrate understanding of the consent form based on a standardized tool administered prior to enrollment. 

**How accurate are the following measures when done as a one-off?: *the estimated fetal weight, due date, and gestational age as calculated by the ultrasound machine are documented in real-time***

 We plan to compare estimated fetal weight on ultrasound to measured birthweight. Estimated due date (and gestational age at delivery) will be calculated using the established ACOG algorithm based on reported last menstrual period unless discrepant with the first ultrasound performed in pregnancy. We acknowledge that gestational age estimation with ultrasound is associated with increasing error as pregnancy advances (although not as much as relying solely on last menstrual period).

**The protocol seems to suggest that the woman has (four or sixteen) sensors placed to measure fetal ECG and maternal vital signs from the time of enrolment (antenatally for some women?) until they are discharged from the hospital. I wasn’t sure if there had been any consideration in the analysis of how having up to 16 sensors placed might impact on the freedom of movement of the mother in labour (and therefore on labour progress) even if they are wireless. I presume that the technology has been tested prior to the study to assess if it is unobtrusive, and robust (for example, in the exertion of labour, and the consequent rise in skin temperature/maternal sweating, do the sensors stay firmly fixed, or do staff have to keep re-siting them, interrupting the women’s concentration on managing her labour?). After birth, if the woman is breastfeeding, it would be useful to know if this impacts the sensors/signals, and how the sensors might impact the success of breastfeeding, if at all. It would be helpful if the final report provides an assessment of the acceptability and feasibility of the sensors during labour and birth and the postpartum period, especially if the proposal is that similar sensors might be used in routine practice in the future for women deemed to be high risk.**

 We agree, the possible impact of the sensors on a woman’s concentration on managing her labor is an important consideration, but unfortunately, we did not enroll women without sensors so are unable to compare this between women with and without sensors during labor. We do plan to publish results of the feasibility of the sensors, which will include data on replacements as well as temporary and permanent removals and associated reasons. The sensors were removed shortly after delivery so did not interfere with breastfeeding. We will acknowledge this as a limitation of our study when results are published.

**I note that the scribes are allocated up to three women simultaneously and that they are supposed to collect a large amount of data in real-time. I wonder if this is actually possible to do with accuracy and completeness. I imagine that this means that up to three women are labouring in one room. If this is indeed the environment in which these data are collected, this needs to be a caveat on the findings, since this kind of context may affect labour progress and outcome. It would be very helpful to have a description of the context and organisational labour room norms in the final report, to establish how far they may or may not have influenced the clinical progress and outcomes of labour and birth. This could include if women were upright and mobile or supine, or in lithotomy, if fundal pressure was used, and if the women had companionship, nutrition, pain relief, and respectful care as recommended by** **WHO** **.**

 We agree – the total volume of participants each scribe was assigned to and their relative acuity at the time will be important data to present in the final report as suggested. We did not (regrettably) explicitly collect individual data on companionship in the labor room and this is a noted limitation of our dataset, especially given the strong recent WHO recommendations. We do collect data on maternal position throughout the intrapartum period both through real-time scribe data and from the physiological sensors, which contain accelerometers. We will report these findings in future publications. Both fundal pressure and pain relief interventions are also collected in real-time by scribes at the bedside.

**The primary outcome measure is a composite. While this makes sense at a population level, it doesn’t really lend itself to a determination about what to do about individual risk. The problem with composite outcomes is that the aetiology and therefore treatment of different components of the composite are very different. While the individual components are included as secondary outcomes, it isn’t clear if there will be sufficient statistical power to establish risk profiles for individuals with these specific outcomes.**

 Since optimal, tailored decision rules are focused on specific decisions, for these hypothesis-generating analyses, we anticipate defining optimality in terms of decision-specific outcomes. Decision-specific outcomes may or may not necessarily be the primary outcome measure, which is intended to capture a summary of the study rather than a targeted goal for a decision rule. With this in mind, our *a priori* sample size considerations for estimating optimal decision rules were based on a generic setting (a binary exposure, a mix of features types, and a binary outcome). We expect to have adequate sample size to learn optimal or near-optimal rules with high probability for a generic setting when the true risk ratio is as low as 1.7 for a binary treatment. From these analyses, we are not targeting the estimation of individual risk profiles, rather we are targeting optimal, tailored treatment decision support among heterogenous individuals. This entails understanding how individuals with heterogeneous features under different treatments respond to those treatments in a rigorous causal framework and learning the optimal boundary between those who should get one treatment rather than another. Based on our extensive simulation studies, data from our study is likely to support finding such optimal boundaries with high probability.

**The sample size calculation section states that the numbers anticipated could provide statistically significant findings for *‘a rare event, one occurring in just 2% of the participants’*'. Presumably then this doesn’t include maternal death. It would, however, probably be sufficient for perinatal mortality in the included countries, though, as noted above, it isn’t clear if these are exclusions for the study, despite the fact that they are included in the composite measure of outcome.**

 Given our sample size, we expect to have adequate precision for outcomes as rare as 2%. This applies to both our composite maternal and neonatal outcomes and the individual maternal morbidity and neonatal morbidity and mortality outcomes used to estimate the composites. We expect maternal deaths to be much rarer than 2% and as such do not expect to have adequate precision to estimate the risk of maternal death as a single individual outcome. However, participants who experience maternal death will be kept in the analysis and contribute to the estimation of the primary maternal composite outcome.

**Conclusion**

 We greatly appreciate your careful review and commentary. We will certainly include plans for sharing with service user organizations and national professional groups and policymakers as part of dissemination activities.

 We agree that systemic factors including organizational and human factors mediate the translation of evidence into clinical practice. The difficulty with translation, generalizability, and transportability is true for evidence generated through a precision analytic lens and also true for evidence generated through more traditional frameworks. Indeed, this issue is not unique to precision medicine.

 We first clarify the role of (optimal) dynamic treatment regimes (DTRs) as evidence. Optimal DTR estimation is a hypothesis-generating goal, a strategy for identifying potential sequences of decision recommendations tailored to individuals that improve outcomes. As with any hypothesis, rigorous testing must be done before broadly applying it into clinical settings. This includes efficacy testing, e.g. a randomized control trial comparing the estimated optimal DTR to standard care. Finally, all evidence relies on thoughtful translation into clinical practice based on contextual feasibility, relevance, and acceptability; precision medicine and DTRs are not an exception, and we certainly would expect care providers and patients to apply precision medicine evidence as it makes sense in specific contexts.

 The statistical precision medicine framework and DTRs allow for a form of data-driven decision support; as we write, they yield treatment recommendations given a set of features. We clarify that DTRs do not make recommendations based on clusters of phenomena associated with particular outcomes. That type of analysis falls within the domain of risk prediction, i.e. prognosis. In contrast, optimal DTR estimation is targeted towards optimal decision support, i.e. prescription of action or treatment, within a rigorous causal inference framework. Consequently, DTRs can delineate optimal treatments for different individuals even if they have the same level of risk. Analytically, this can be described as identifying phenotypic subgroups (heterogeneity) and how those subgroups interact with different feasible treatments, as opposed to how they associate with outcomes, at each decision point (see Kosorok and Laber (2019) ^1^ for a discussion of this difference). The analytic framework enables understanding optimal sequences of treatment that account for long-term outcomes (as opposed to myopic gains at the cost of long-term benefits overall) where each decision rule (one for each decision point) “takes in” individual features and “gives out” an optimal treatment recommendation based on the history and features of that particular individual. From a population perspective, improving treatment decisions for individuals yields population-level improvements (or at least no worse) in outcomes over the one-size-fits-all approach of following the same protocol for all individuals faced with the same decision without regard to individual features, history, and response to past treatment.

 The precision medicine framework is well-poised to address some of the concerns regarding systemic factors that affect health outcomes. Because the framework leverages heterogeneity, structural differences between settings can be addressed through DTR estimation. With the substantial breadth of data collected in this study, which includes participants in 4 hospitals across 3 countries, we have a unique opportunity to leverage differences in setting in our planned analyses—that is, we can include site differences as part of algorithmic recommendations. Additionally, methods for transporting causal inferences from one setting to another is an active area of research with a number of methods and strategies already available for use. ^2^

 Of course, purely statistical methods are not a panacea, and are but a starting place for understanding the optimal use of precision medicine evidence at scale in real-world settings. Bringing evidence to bear in complex systems falls into multiple domains including implementation science, systems science, statistical science, and clinical science, among others. These scientific domains must also incorporate key stakeholder engagement to optimize feasibility, relevance, acceptability, algorithmic fairness, equity, access, and to minimize potential harms. One strategy for this type of engagement is systems-aligned precision medicine, a general framework for embedding a systems lens to the translation of precision medicine evidence to clinical contexts. ^3^  As we move forward, we certainly intend to bring a systems-aware lens to the application of any hypothesis generated from our analyses and carefully test our hypotheses before deploying any decision support tool at scale. The data we have generated from this study and our planned analyses are a starting point along the path of clinical decision support tools from optimal precision treatment algorithms towards scaled implementation.

**References**

 1.         Kosorok MR, Laber EB. Precision Medicine. Annu Rev Stat Appl 2019;6:263-86.

 2.         Degtiar I, Rose S. A Review of Generalizability and Transportability. Annual Review of Statistics and Its Application 2023;10:null.

 3.         Kahkoska AR, Freeman NLB, Hassmiller Lich K. Systems-Aligned Precision Medicine—Building an Evidence Base for Individuals Within Complex Systems. JAMA Health Forum 2022;3:e222334-e.

# Peer review: Reviewer response for version 1

**DOI:** 10.21956/gatesopenres.15003.r32416

**Published:** 2022-09-06

**Authors:** G. Justus Hofmeyr

**Affiliations:** 1 Effective Care Research Unit, Eastern Cape Department of Health, Universities of the Witwatersrand and Walter Sisulu, East London, South Africa; 2 University of Botswana, Gaborone, Botswana

This paper describes the study protocol for a large, multi-country observational study focused on risk factors, interventions, and outcomes related to labour and the early postpartum period. A unique feature is detailed blinded physiological data acquisition, which in my opinion has the potential to provide important information. The planned statistical analysis is complex - I am not familiar with many of the statistical procedures and therefore not able to comment on them.

 The following suggested additional information may be of interest to readers:

1. What clinical fetal monitoring unblinded to caregivers has been employed?
2. Has information been collected on the presence of childbirth companions, which may impact several of the outcomes being measured, such as low Apgar scores?
3. Has information been collected on maternal position, particularly the supine position during the second stage of labour, and will it be possible to relate maternal position to the physiological data collected (or to impute maternal position from the data provided by the sensors)?
4. Were partographs used for guiding labor management, and if so which design of partograph?
5. Were clinical management protocols in use? If so, some information on the protocols would be useful.
6. It would be useful to update certain statements, for example, whether recruitment, which is due to have been completed, has indeed been completed, and whether certain proposed changes in the technology available did in fact take place.
7. Study participants: the phrase: "3) admission to hospital labor and delivery unit with plan for either (a) vaginal delivery or (b) cesarean delivery in the presence of signs/symptoms of parturition, elevated blood pressure, or fever;" is not very clear - perhaps it could be written more precisely.

Is the study design appropriate for the research question?

Yes

Is the rationale for, and objectives of, the study clearly described?

Yes

Are sufficient details of the methods provided to allow replication by others?

Partly

Are the datasets clearly presented in a useable and accessible format?

Not applicable

Reviewer Expertise:

General obstetrics including hypertensive disorders, postpartum hemorrhage and intrapartum care

I confirm that I have read this submission and believe that I have an appropriate level of expertise to confirm that it is of an acceptable scientific standard.

# Response

**Published:** 2022-12-05

**Authors:** Joan Price

Thank you for your careful review and for approving our manuscript for publication. Please see point-by-point responses to your questions and comments below.

**What clinical fetal monitoring unblinded to caregivers has been employed?**

 Standard-of-care fetal monitoring at each of our four sites relies on intermittent monitoring by fetoscope or by handheld Doppler. Periodically, high-risk cases are monitored by continuous monitoring primarily at the Belgaum site. Study sensors were not intended for clinical care, so no sensor data was provided to caregivers in real time. See revised manuscript “sensor placement” page 6.

**Has information been collected on the presence of childbirth companions, which may impact several of the outcomes being measured, such as low Apgar scores?**

 We did not collect this information, but anecdotally childbirth companions are rarely present at any of the study sites.

**Has information been collected on maternal position, particularly the supine position during the second stage of labour, and will it be possible to relate maternal position to the physiological data collected (or to impute maternal position from the data provided by the sensors)?**

 We do collect information on maternal position (and position changes). We plan to validate positional data from the maternal sensors using real-time data collected by scribes at the bedside. See revised section on “sensor placement” page 6.

**Were partographs used for guiding labor management, and if so which design of partograph?**

 Yes, partographs are regularly used to guide labor management at each site. At all four study sites, the modified WHO partograph is used. The most recent WHO Labour Care Guide has not yet been implemented at any of the study sites.

**Were clinical management protocols in use? If so, some information on the protocols would be useful.**

 Clinical care at each site is guided by clinical protocols and algorithms for the management of various conditions such as hypertension, preeclampsia, antepartum hemorrhage, postpartum hemorrhage, etc. Our study scribes document care received (i.e., actions, diagnoses, medications) in real time, but do not assess providers’ adherence to or variance from established clinical management protocols per se. We plan to investigate both variations in care between sites and variance from established protocols in future manuscripts, but think that a description of the various protocols at 4 different sites is beyond the scope of this protocol paper.

**It would be useful to update certain statements, for example, whether recruitment, which is due to have been completed, has indeed been completed, and whether certain proposed changes in the technology available did in fact take place.**

 As we write, recruitment has indeed concluded, and we transitioned from Meridian to wireless Sibel fetal sensors as planned. These statements have been updated in the revised manuscript on pages 6 and 13.

**Study participants: the phrase: "3) admission to hospital labor and delivery unit with plan for either (a) vaginal delivery or (b) cesarean delivery in the presence of signs/symptoms of parturition, elevated blood pressure, or fever;" is not very clear - perhaps it could be written more precisely.**

 This is the eligibility criterion as written verbatim in the protocol. We have clarified this further in the manuscript.
